# Smoking Status and HbA1c in U.S. Adults Without Diabetes: A Cross-Sectional National Health and Nutrition Examination Survey (NHANES) Analysis

**DOI:** 10.7759/cureus.108658

**Published:** 2026-05-11

**Authors:** Chibuzo C Manafa, Patrick O Manafa, Nnamdi Okoli, Chukwuzitelu Okafor-Udah, Stephen Adilih, Ngozi Ogo, Ndidi-amaka Adilih

**Affiliations:** 1 Family Medicine, Queensland Medical Clinic, Calgary, CAN; 2 Clinical Chemistry, Nnamdi Azikiwe University, Nnewi, NGA; 3 Family Medicine, University of Liverpool Hospital Group, Liverpool, GBR; 4 Family Medicine, Fivecees Medical Center, Calgary, CAN

**Keywords:** cotinine, diabetes, epidemiology, hba1c, smoking cessation

## Abstract

Background: Cigarette smoking is a recognized risk factor for type 2 diabetes, and prior work has reported higher glycated hemoglobin (HbA1c) levels among smokers. Whether this reflects a direct metabolic effect of smoking or residual confounding by central adiposity, systemic inflammation, and lipid dysregulation remains unclear, and whether the association persists after cessation in adults without diabetes has not been fully characterized.

Aim: We examined associations between smoking and HbA1c among United States (U.S.) adults, and whether these associations vary by diabetes status.

Methods: We analyzed National Health and Nutrition Examination Survey (NHANES) data from 2015-2018 for adults aged ≥20 years. Smoking was assessed by self-report and serum cotinine. Survey-weighted multivariable linear regression was used to evaluate the association between smoking and HbA1c in the full population (N=9,214) and in adults without diabetes (N=7,328), adjusting for demographics, blood pressure, waist circumference, lipids, and high-sensitivity C-reactive protein (hsCRP).

Results: After adjustment for cardiometabolic covariates, there was no statistically significant association between smoking and HbA1c in the full population (former: β=0.029%, p=0.30; current: β=0.053%, p=0.13). Among adults without diabetes, former smoking was not associated with HbA1c, whereas current smoking remained significantly associated (former: β=−0.001%, p=0.923; current: β=0.067%, p<0.001). These findings were similar when cotinine was used as the exposure measure, with active smoking (≥3.0 ng/mL) associated with higher HbA1c among non-diabetic adults (p<0.001), but not in the full population.

Conclusions: Among adults without diabetes, current smoking was statistically significantly associated with higher HbA1c, whereas former smoking was not. The formal smoking × diabetes interaction test did not reach statistical significance, so the subgroup pattern should be interpreted as hypothesis-generating. The absence of a significant association in former smokers is compatible with attenuation following cessation. These findings may inform cessation counseling and diabetes screening and warrant replication in larger, adequately powered studies.

## Introduction

Cigarette smoking is a well-known risk factor for type 2 diabetes mellitus, and meta-analyses have noted an increased risk of about 30-50% among current smokers when compared to never smokers [1,2]. Previous studies have consistently reported higher HbA1c levels among smokers [3-5]. However, the biological pathways underlying this association and its clinical implications remain incompletely understood.

Glycated hemoglobin (HbA1c) is a measure of average glycemia over 2-3 months, corresponding to the lifespan of red blood cells. Factors other than glucose concentration, such as red blood cell turnover, hemoglobin characteristics, systemic inflammation, and adiposity, may affect HbA1c levels [6,7]. Since diabetes screening measures rely on strict HbA1c thresholds of 5.7% for prediabetes and 6.5% for diabetes [8], a modest increase in HbA1c could influence risk classification; therefore, clarifying the mechanisms underlying the association between smoking and HbA1c is of potential clinical and epidemiological relevance.

The association between smoking and HbA1c may differ based on an individual’s diabetes status, and comparing current with former smokers can help differentiate reversible from more long-term effects. Similar HbA1c levels in former and never smokers would support reversibility of smoking's metabolic effects on glycemia, consistent with recovery of insulin signaling following cessation [6]. However, persistently elevated levels following smoking cessation could suggest longer-lasting metabolic effects targeting systemic inflammation and adiposity as proposed by Chiolero et al [7].

In their study of individuals with established diabetes, Kar et al. reported that glycemic recovery following cessation may be incomplete and may require several years of sustained abstinence [9]. However, it is still unclear whether similar patterns of association or reversibility are present in adults without diabetes.

Previous studies have mostly relied on self-reported smoking status, but this can be subject to misclassification and social desirability bias [10]. Serum cotinine, the primary metabolite of nicotine with a half-life of 16-18 hours, serves as an objective biomarker of recent tobacco exposure and can be an alternative measure [11]. Evaluating both self-reported smoking status and cotinine concentrations within the same analytical framework may help distinguish between exposure misclassification and true biological associations.

Findings by Clair et al. showed that cotinine was associated with higher HbA1c levels among non-diabetic adults [3]. Given the declining smoking prevalence in recent years [12], the present analysis builds on this foundational work by examining recent National Health and Nutrition Examination Survey (NHANES) cycles.

Using NHANES 2015-2018 data, we examined associations of smoking with HbA1c in U.S. adults. Our primary objective was to evaluate whether current and former smoking, relative to never smoking, show differential associations with HbA1c among adults without diabetes, after comprehensive adjustment for demographic and cardiometabolic confounders. We hypothesized that current smokers would demonstrate higher HbA1c than never smokers, whereas former smokers would demonstrate levels comparable to never smokers, consistent with at least partial metabolic reversibility following cessation. Secondary objectives were to (i) compare effect estimates derived from self-reported smoking status and serum cotinine within the same analytical framework, and (ii) formally test for effect modification by diabetes status. We expected the self-report and cotinine-based estimates to be concordant and the attenuation pattern observed in the full population to be largely explained by adiposity, inflammation, and lipid profiles.

## Materials and methods

Study design and data source

We conducted a cross-sectional analysis of data from the NHANES, a nationally representative survey of the noninstitutionalized U.S. population. We pooled two continuous cycles (2015-2016 and 2017-2018) because these were the most recent pre-pandemic cycles in which serum cotinine, hsCRP, and HbA1c were measured using consistent laboratory methods. The 2019-2020 cycle was not used because data collection was truncated by the COVID-19 pandemic, and the resulting file is released as a combined 2017-March 2020 pre-pandemic dataset with altered weighting, which would have complicated pooling. Earlier cycles used different hsCRP assay methods and were therefore not directly combinable with the 2015-2018 cycles.

NHANES protocols were approved by the National Center for Health Statistics (NCHS) Research Ethics Review Board, and all participants provided written informed consent. The present study used publicly available, de-identified data and was therefore exempt from additional institutional review board review.

Study population

We restricted participants to adults aged ≥20 years. The initial sample across the four years included 19,225 participants. We pre-specified a complete-case analytic strategy: analyses were restricted to individuals with complete data on HbA1c, smoking status, waist circumference, blood pressure, lipids, and hsCRP. This approach was pre-specified at the analysis plan stage on the basis of low expected missingness, which proved to be 2.3-5.8% overall and varied by less than 3% across smoking categories (Appendix 1), supporting the missing-at-random assumption and making complete-case analysis an efficient and unbiased choice. The analytic samples differ slightly across exposure definitions because cotinine and self-reported smoking have non-identical patterns of non-missingness: N = 9,214 for self-reported smoking analyses, N = 9,221 for cotinine-based analyses, and N = 7,328 for the non-diabetic subgroup analyses.

Exposure assessment

Self-Reported Smoking

Smoking status was determined using standardized NHANES questionnaire items (SMQ020 and SMQ040) from the Smoking - Cigarette Use (SMQ) questionnaire [13]. Participants who had smoked <100 cigarettes in their lifetime were classed as never smokers, and they were used as the reference group. We grouped those participants who had smoked ≥100 cigarettes in their lifetime but are not currently smoking as former smokers, while those who had smoked ≥100 cigarettes in their lifetime and still continue to smoke were classed as current smokers. 

Serum Cotinine

Serum cotinine concentrations were quantified using isotope-dilution high-performance liquid chromatography coupled with tandem mass spectrometry. Cotinine is a major metabolite of nicotine and is used as a biomarker of recent tobacco exposure. The limit of detection (LOD) was 0.011 ng/mL.

For the main analyses, cotinine levels were categorized into three pre-specified groups based on established biomarker thresholds [14,15]: <0.05 ng/mL (essentially unexposed), 0.05-<3.0 ng/mL (environmental tobacco smoke [ETS] exposure), and ≥3.0 ng/mL (active smoking). The ≥3.0 ng/mL threshold is the widely accepted cut point for distinguishing active tobacco use from non-use in U.S. adults [15], while 0.05 ng/mL is a conventional lower bound separating ETS-exposed individuals from the essentially unexposed [14]. Serum cotinine values reported below the LOD were retained at the LOD as provided in the NHANES public-use files; approximately 34% of values clustered at this limit. Because this clustering makes an unmodified continuous analysis unstable, the primary analysis used the pre-specified categorical cut points, and a quintile analysis was reported as the primary dose-response sensitivity check. A log-transformed continuous analysis and a winsorized analysis (capped at the 99th percentile) were additionally conducted to confirm robustness to specification choices (Appendices 2-3).

Concordance between self-reported smoking and cotinine categories was assessed using survey-weighted cross-tabulation.

Outcome assessment

Our primary outcome, HbA1c, was analyzed as a continuous variable. HbA1c was measured using high-performance liquid chromatography from whole blood.

Covariates

Covariates were selected a priori based on known confounders of the smoking-HbA1c relationship. Age, sex, and race/ethnicity (RIDRETH3) capture demographic confounding and were included in all models. The cardiometabolic covariates--systolic blood pressure (SBP; the mean of readings 2-4), waist circumference, non-HDL cholesterol (total cholesterol minus HDL cholesterol), and hsCRP--are established components of the cardiometabolic syndrome and are each independently associated with both smoking behavior and glycemia, placing them on the causal pathway of interest. hsCRP showed a right-skewed distribution and was modeled as log(1 + hsCRP).

BMI and waist circumference are strongly correlated, and we therefore did not include both in the primary model. We instead compared models containing BMI alone (Model 3), waist circumference alone (Model 4), and both together (Model 5). Waist circumference was retained as the primary adiposity measure because it more closely reflects central adiposity, which has been shown to correlate more strongly with cardiometabolic risk than BMI [16].

Diabetes Definition and Subgroup Analysis

Diabetes was defined based on self-reported data or HbA1c. Participants were considered to have diabetes if they had been told by a health professional that they had diabetes or if the HbA1c was ≥6.5%.

For adults without diabetes, we repeated the primary model, restricting it to participants who had not been told by a health professional that they had diabetes and those with HbA1c <6.5% (N=7,328).

Statistical analysis

All analyses accounted for NHANES’ complex survey design using examination weights (WTMEC2YR), primary sampling units (PSUs; SDMVPSU), and strata (SDMVSTRA). All data were analyzed using R version 4.5.2 (R Foundation for Statistical Computing, Vienna, Austria). Following the recommendations by the National Center for Health Statistics, four-year weights were constructed by dividing the 2-year examination weights by 2. Variance estimation used Taylor series linearization with adjustment for strata containing a single primary sampling unit.

We built a hierarchical sequence of survey-weighted linear regression models to isolate the incremental contribution of each confounder domain to the smoking-HbA1c association. Model 1 adjusted for demographics alone (age, sex, race/ethnicity) to establish the unadjusted demographic base. Model 2 added systolic blood pressure. Models 3 and 4 separately examined BMI and waist circumference (each built on Model 2) to compare general versus central adiposity as candidate pathways. Model 5 included both measures simultaneously to evaluate collinearity and confirm which adiposity metric carried the primary signal. Model 6 was prespecified as the primary model because it incorporates the full prespecified set of cardiometabolic confounders--systolic blood pressure, waist circumference, non-HDL cholesterol, and log(1 + hsCRP)--without introducing the additional missingness associated with socioeconomic variables. Model 7 additionally adjusted for education and poverty-income ratio and is retained as a sensitivity model given its reduced sample size (N = 8,151) due to missing socioeconomic data. For models with BMI included, the sample size was slightly smaller (N = 9,198) due to missing anthropometric data.

Coefficient-level significance in the survey-weighted linear regression models was assessed using Wald tests based on design-adjusted (Taylor series linearization) standard errors. The smoking × diabetes interaction was tested by including a product term in the fully adjusted model and was evaluated with a survey-weighted Wald test on the interaction coefficients. The trend across cotinine quintiles was tested by assigning the median cotinine value of each quintile and entering this as a continuous term in the fully adjusted model. Baseline characteristics were compared across smoking categories using survey-weighted means (continuous variables) and survey-weighted proportions (categorical variables), with standard errors derived from Taylor series linearization.

Statistical significance was defined as a two-sided p<0.05.

Transparency and Reproducibility

The R analysis script and session information are available from the corresponding author upon reasonable request. All data used in this study are publicly available through the National Health and Nutrition Examination Survey (NHANES) at https://www.cdc.gov/nchs/nhanes/.

## Results

Study population characteristics

**Figure 1:**
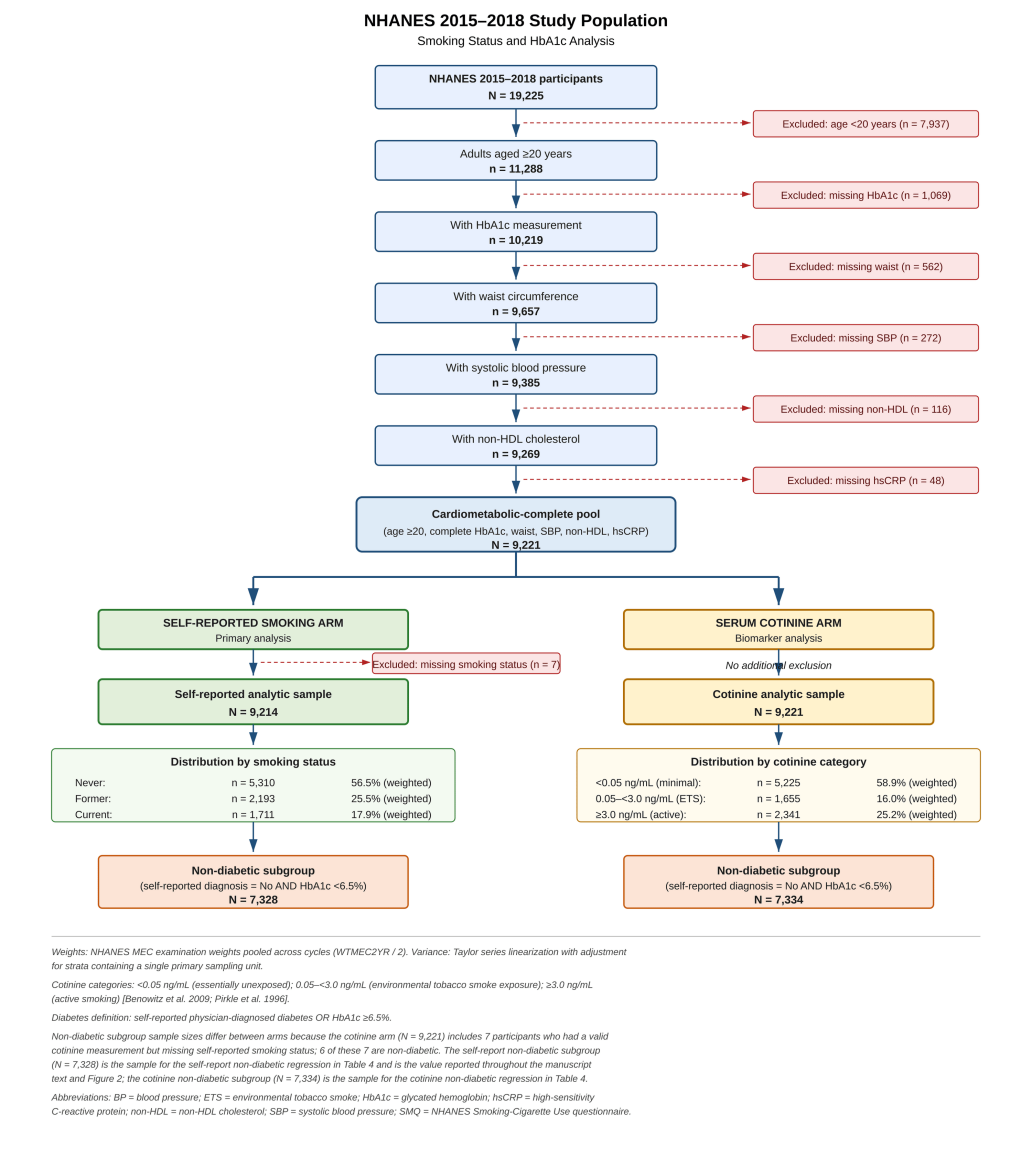
Study Flow Diagram Participant flow diagram for NHANES 2015-2018 analytic samples. N = 7,328 is the non-diabetic subgroup within the self-report arm (n = 9,214). N = 7,334 is the non-diabetic subgroup within the cotinine arm (n = 9,221). The cotinine arm includes 7 additional participants who had a valid serum cotinine measurement but missing self-reported smoking status; 6 of those 7 are non-diabetic, accounting for the difference.

From an initial sample of 19,225 NHANES participants, we excluded participants aged <20 years. Among the remaining participants, we further excluded those with missing data (Figure 1). A total of 9,214 adults were included in the self-reported smoking analyses, 9,221 in the cotinine analyses, and 7,328 adults without diabetes in the subgroup analyses. Missingness across variables was low (2.3-5.8%) and similar across smoking groups. All analyses incorporated NHANES survey weights.

Weighted prevalence of smoking categories was 56.5% never, 25.5% former, and 17.9% current smokers (Figure 1). Current smokers were younger (mean age 44.3 years) than never smokers (46.8 years) and former smokers (53.7 years) and had lower educational attainment. Mean HbA1c values were 5.62% (SE 0.02), 5.79% (SE 0.03), and 5.65% (SE 0.03) among never, former, and current smokers, respectively. Former smokers had greater waist circumference and systolic blood pressure than the other groups. Former and current smokers were more likely to be male compared with never smokers (Table 1).

**Table 1:** Baseline Characteristics by Self-Reported Smoking Status (N=9,214) Abbreviations: BMI, body mass index; HbA1c, hemoglobin A1c; HDL, high-density lipoprotein; BP, blood pressure. Values are weighted means (SE) or percentages. Percentages sum to 100% within smoking category.

Characteristic	Never	Former	Current
Continuous variables - weighted mean (SE)			
Age, years	46.80 (0.43)	53.67 (0.68)	44.34 (0.67)
HbA1c, %	5.62 (0.02)	5.79 (0.03)	5.65 (0.03)
BMI, kg/m²	29.41 (0.21)	30.50 (0.24)	28.84 (0.25)
Waist circumference, cm	99.22 (0.53)	105.09 (0.60)	99.73 (0.69)
Systolic BP, mmHg	122.30 (0.36)	126.21 (0.69)	122.52 (0.76)
Diastolic BP, mmHg	71.36 (0.39)	71.24 (0.52)	71.59 (0.64)
Non-HDL cholesterol, mg/dL	134.26 (1.11)	139.34 (1.79)	139.29 (1.79)
High sensitivity C-reactive protein, mg/L	3.55 (0.13)	3.97 (0.21)	4.31 (0.24)
Categorical variables - weighted %			
Sex			
Male	41.4%	60.9%	54.4%
Female	58.6%	39.1%	45.6%
Race/ethnicity			
Mexican American	10.3%	7.0%	7.3%
Other Hispanic	7.4%	5.4%	5.0%
Non-Hispanic White	60.4%	73.3%	64.1%
Non-Hispanic Black	11.3%	6.9%	13.6%
Non-Hispanic Asian	7.7%	2.7%	2.5%
Other/multiracial	2.9%	4.7%	7.5%
Education			
Less than high school	10.4%	12.0%	18.2%
High school / GED	20.1%	26.2%	34.8%
Some college	30.0%	34.8%	34.6%
College graduate or higher	39.4%	27.1%	12.5%

Self-reported smoking status and HbA1c

In Model 1, adjusted for only demographics, both former and current smoking were associated with higher HbA1c compared with never smoking (former: β=0.078%, 95% CI 0.016 to 0.140; current: β=0.077%, 95% CI 0.018 to 0.135). When adjusted for systolic blood pressure, there was minimal change in effect estimates (Model 2). The addition of BMI reduced the association for former smoking but not for current smoking. However, after the inclusion of waist circumference, non-HDL cholesterol, and log(1+hsCRP) as shown in model 6, effect estimates decreased and were no longer statistically significant (former: β=0.029%, 95% CI -0.028 to 0.085; current: β=0.053%, 95% CI -0.016 to 0.121).

Model 7 was added as a sensitivity model as it had fewer participants due to missing values (n=8151). It adjusted for education and poverty-income ratio but did not materially change these findings (Table 2). Age, waist circumference, non-HDL cholesterol, and log(1+hsCRP) remained independently associated with HbA1c (Table 3).

**Table 2:** Sequential Models - Association Between Self-Reported Smoking Status and HbA1c (N=9,214) Abbreviations: BMI, body mass index; CI, confidence interval; HbA1c, hemoglobin A1c; HDL, high-density lipoprotein; hsCRP, high-sensitivity C-reactive protein; PIR, poverty–income ratio; SBP, systolic blood pressure. Note: β coefficients represent adjusted differences in HbA1c (%) relative to never smokers. Model 6 is the primary fully adjusted model. Model 7 includes education and PIR; N=8,151 due to missing socioeconomic data (complete-case analysis). Never smokers are the reference group.

Model	Covariates	Former vs Never β (95% CI)	p	Current vs Never β (95% CI)	p
M1	Age + Sex + Race/Ethnicity	0.078 (0.016, 0.140)	0.016	0.077 (0.018, 0.135)	0.012
M2	Model 1 + SBP	0.075 (0.011, 0.138)	0.024	0.073 (0.015, 0.130)	0.016
M3	Model 2 + BMI	0.048 (-0.013, 0.109)	0.113	0.093 (0.030, 0.155)	0.006
M4	Model 2 + waist circumference	0.035 (-0.024, 0.094)	0.233	0.078 (0.016, 0.140)	0.017
M5	Model 2 + BMI + waist circumference	0.033 (-0.026, 0.093)	0.257	0.077 (0.011, 0.142)	0.025
M6	Model 4 + non-HDL + log(1 + hsCRP)	0.029 (-0.028, 0.085)	0.301	0.053 (-0.016, 0.121)	0.125
M7	Model 6 + education + PIR	0.028 (-0.037, 0.093)	0.363	0.033 (-0.049, 0.116)	0.398

**Table 3:** Complete Regression Output for Model 6 (All Race/Ethnicity Categories) Abbreviations: CI, confidence interval; HbA1c, hemoglobin A1c; HDL, high-density lipoprotein; SE, standard error; hsCRP, high-sensitivity C-reactive protein. Footnote: β coefficients represent adjusted differences in HbA1c (%). Reference groups: never smokers, male sex, Mexican American race/ethnicity.

Covariate	β	95% CI	SE	p-value
Smoking status				
Former smoker (vs never)	0.0286	-0.0280, 0.0853	0.0268	0.301
Current smoker (vs never)	0.0525	-0.0161, 0.1212	0.0326	0.125
Demographics				
Age (per year)	0.0155	0.0141, 0.0168	0.0006	<0.001
Sex				
Female (vs male)	-0.0651	-0.1141, -0.0161	0.0232	0.012
Race/ethnicity				
Other Hispanic (vs Mexican American)	-0.1299	-0.2728, 0.0131	0.0678	0.072
Non-Hispanic White (vs Mexican American)	-0.3519	-0.4899, -0.2140	0.0654	<0.001
Non-Hispanic Black (vs Mexican American)	-0.0055	-0.1329, 0.1220	0.0604	0.929
Non-Hispanic Asian (vs Mexican American)	0.0285	-0.1211, 0.1781	0.0709	0.693
Other/multiracial (vs Mexican American)	-0.2187	-0.4011, -0.0363	0.0865	0.022
Cardiometabolic factors				
Systolic blood pressure (per mmHg)	0.0016	-0.0004, 0.0036	0.0010	0.106
Waist circumference (per cm)	0.0085	0.0068, 0.0103	0.0008	<0.001
Non-HDL cholesterol (per mg/dL)	0.0009	0.0003, 0.0014	0.0003	0.007
log(1 + hsCRP)	0.1412	0.1062, 0.1762	0.0166	<0.001

Associations by exposure measure and diabetes status

**Figure 2:**
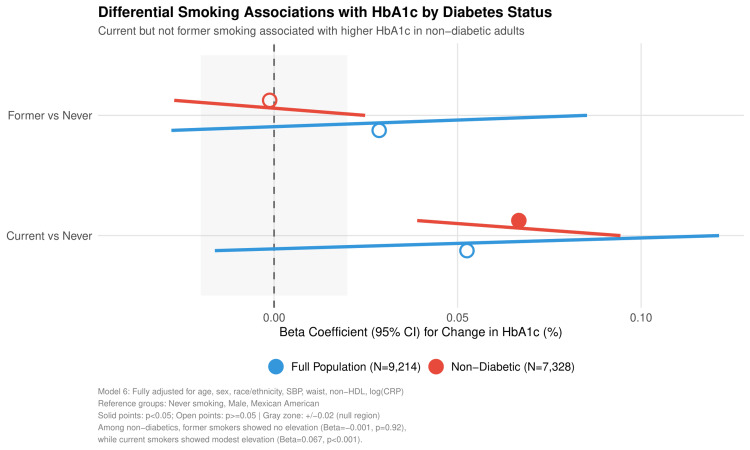
Differential Smoking Associations With HbA1c by Diabetes Status Comparison of smoking associations with HbA1c in full population (N=9,214) versus participants without diabetes (N=7,328). Effect estimates from fully adjusted Model 6. Diabetes defined as self-reported diagnosis or HbA1c ≥6.5%. Solid points: p<0.05; open points: p≥0.05. Gray zone: ±0.02% HbA1c (approximate null region).

After full adjustment in the overall population, there was no statistically significant association between smoking and HbA1c, and this was true for both self-reported smoking and cotinine-defined exposure (Table 4). However, among participants without diabetes (n=7,328), current smoking was associated with higher HbA1c (β=0.067%, 95% CI 0.039 to 0.094, p<0.001), whereas former smoking was not (β=-0.001%, 95% CI -0.027 to 0.025, p=0.923). Associations did not show a significant difference by diabetes status (former p=0.375; current p=0.808). Also, higher HbA1c was observed among participants with cotinine levels consistent with active smoking (≥3.0 ng/mL), but not among those showing environmental exposure levels (β=0.064%, 95% CI 0.036 to 0.093, p<0.001) (Table 4, Figure 2).

**Table 4:** Association Between Smoking and HbA1c by Exposure Measure and Diabetes Status Abbreviations: HbA1c, hemoglobin A1c; ETS, environmental tobacco smoke; CI, confidence interval. Footnote: β coefficients represent percentage-point differences in HbA1c from fully adjusted models. Sample sizes: N=9,214 (self-reported smoking), N=9,221 (serum cotinine), N=7,328 (self-report non-diabetic subsample), N=7,334 (cotinine non-diabetic subsample).

Exposure	Full Population	P-value	Non-Diabetic Adults	P-value
	β (95% CI)		β (95% CI)	
Self-reported smoking status				
Former vs never	0.029 (-0.028, 0.085)	0.301	-0.001 (-0.027, 0.025)	0.923
Current vs never	0.053 (-0.016, 0.121)	0.125	0.067 (0.039, 0.094)	<0.001
Serum cotinine categories				
0.05–<3.0 ng/mL (ETS) vs <0.05	0.004 (-0.053, 0.062)	0.880	0.021 (-0.007, 0.048)	0.130
≥3.0 ng/mL (active) vs <0.05	0.036 (-0.037, 0.108)	0.313	0.064 (0.036, 0.093)	<0.001

Approximately one-third of cotinine values (34.2%) were at the detection limit (0.011 ng/mL). Regardless of how cotinine was modeled--whether by categories, quintiles, or as a continuous log-transformed variable--no monotonic dose-response relationship with HbA1c was observed (p for trend=0.217). Estimates were similar after winsorization at the 99th percentile (Appendices 2-3).

Concordance between self-reported smoking and serum cotinine

There was high overall concordance between self-reported smoking and cotinine categories. Cotinine levels consistent with active smoking were observed in 6.1% of never smokers and 18.1% of former smokers (Appendix 4).

## Discussion

In this study, we analyzed NHANES data from 2015-2018. This represents an ethnically diverse cohort of U.S. adults. We found that the association between smoking and HbA1c was largely attenuated after comprehensive adjustment for central adiposity, inflammatory markers, and lipid measures in the full population. Further subgroup analyses showed that among participants without diabetes, there was a modest rise in HbA1c for current smokers, while former smokers had similar values to never smokers.

**Figure 3:**
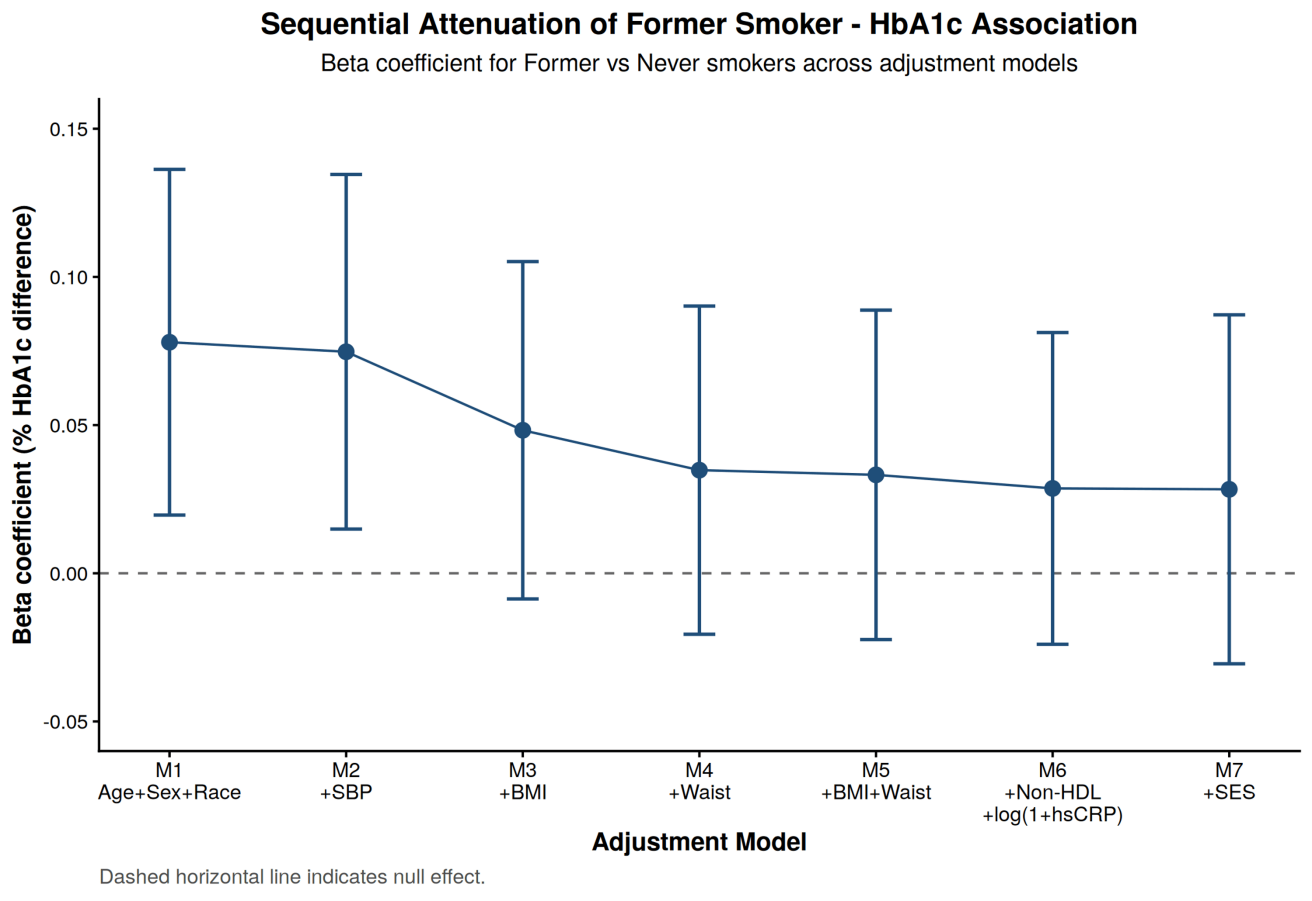
Sequential Attenuation of Former Smoker-HbA1c Associations Across Adjustment Models β coefficients (95% CIs) for former vs. never smoker comparison across seven hierarchical models. Dashed line indicates null effect.

In a prior NHANES analysis, Clair et al. reported a positive association between smoking and HbA1c after adjustment for multiple covariates [3]. Similar findings were observed in the EPIC-Norfolk study and in analyses of the Korea National Health and Nutrition Examination Survey [4,5]. However, these studies did not adjust simultaneously for adiposity, inflammatory markers, and lipid profiles. To contextualise this difference, we reconstructed adjustment strategies similar to those in previous studies. Our Model 4, which included demographics, blood pressure, and waist circumference, mirrored previous reports and yielded similar findings. Once inflammatory indices and lipid measures were added, the association attenuated further and was no longer statistically significant. We observed a decrease of approximately 63% in the coefficient for former smokers and about one-third for current smokers from the base model to the fully adjusted model (Model 6) (Figure 3). 

This pattern suggests that the associations observed in minimally adjusted models may reflect clustering of metabolic and inflammatory pathways rather than a direct, independent effect of smoking on glycemia.

Adjustment for waist circumference attenuated the association more than BMI, and the model including both measures yielded estimates similar to the waist circumference-adjusted model. This suggests that central adiposity, as measured by waist circumference, is the primary adiposity-related pathway. For current smokers, adjusting for BMI alone increased the coefficient, consistent with the lower body weight commonly observed in active smokers [7].

In the subgroup analyses, after full adjustment for blood pressure, adiposity, inflammatory markers, and lipid measures, HbA1c levels among former smokers were similar to those of never smokers. This pattern suggests that the effect of smoking on HbA1c may diminish following cessation, although the cross-sectional design does not allow us to determine timing or direction. This pattern was observed despite former smokers having higher adiposity than never smokers (BMI 30.50 vs. 29.41 kg/m²; waist circumference 105.09 vs. 99.22 cm). These findings were consistent when cotinine concentrations were used as the exposure metric (β=0.064%, p<0.001).

The formal smoking × diabetes interaction test did not reach statistical significance (former p = 0.375; current p = 0.808). Accordingly, the subgroup differences should be interpreted as hypothesis-generating rather than as evidence of effect modification. Nonetheless, the observed pattern - a statistically significant association among current smokers without diabetes and a null association among former smokers without diabetes - is compatible with known biological mechanisms of smoking-induced insulin resistance and its partial reversibility [6], and warrants replication in larger, adequately powered studies.

We observed a modest discordance between self-reported smoking and cotinine categories. This could be as a result of relapse, intermittent smoking, or social desirability bias [10]. Nevertheless, findings were consistent across both exposure definitions. In the non-diabetic subgroup, the cotinine-based and self-report-based findings were closely concordant (active cotinine ≥3.0 ng/mL: β = 0.064%, p < 0.001; current self-reported smoking: β = 0.067%, p < 0.001), while the ETS category (0.05-<3.0 ng/mL) was not statistically significantly associated with HbA1c. Quintile and log-transformed cotinine analyses showed no monotonic dose-response (p for trend = 0.217). Together, these patterns are compatible with a threshold effect at active-smoking levels rather than a graded effect across the full range of nicotine exposure.

Our finding contrasts with longitudinal evidence from populations with established diabetes. Kar et al., in a meta-analysis of 98,978 individuals with diabetes, reported HbA1c levels that were 0.61% higher in current smokers than in non-smokers, noting that it may take up to 10 years after quitting for HbA1c levels to converge [9]. Similarly, Lycett et al. described an initial worsening of HbA1c among smokers with diabetes during the first year after cessation [17]. In contrast, we did not observe residual elevation among non-diabetic former smokers, suggesting that smoking-associated differences in HbA1c may vary by diabetes status, although this requires confirmation in longitudinal studies.

Several mechanisms may contribute to these observations. Smoking induces systemic inflammation and oxidative stress, impairing insulin signaling and promoting insulin resistance [18,19]. Our study found that the greatest attenuation occurred after adjusting for central adiposity. Waist circumference alone was associated with approximately 58% of the observed attenuation in the former smoking group. Non-HDL cholesterol and hsCRP led to further attenuation, although to a lesser degree than adiposity. HsCRP also demonstrated the strongest independent association with HbA1c among all covariates in the fully adjusted model (β=0.141, p<0.001), suggesting that inflammation is strongly associated with glycemia, despite its effect being secondary to visceral adiposity. Of note, hsCRP levels in former smokers remained modestly higher than those of never smokers, raising the possibility that improvements in glycemia may occur despite residual low-grade inflammation. This also mirrors several prior reports suggesting that inflammatory response resolution may lag behind after smoking cessation, even when other metabolic parameters have stabilized [20-22].

Functional β-cells in early phases of metabolic dysfunction may trigger insulin release to counter some of the effects of smoking on glycemia. This may explain the mild hyperglycemia observed despite hyperinsulinemia in early insulin resistance [23]. Bergman et al., in their experimental work, demonstrated that smoking-induced insulin resistance operates primarily by impairing downstream insulin signaling, which in turn reverses partially within weeks of abstaining from smoking [6]. Given that HbA1c measures average glycemia over 2-3 months, even partial recovery of insulin sensitivity over that period may be enough to reduce HbA1c levels despite post-cessation weight gain, explaining the absence of HbA1c elevation observed in non-diabetic former smokers.

The magnitude of the HbA1c difference observed among current smokers without diabetes (β = 0.067%) is modest at the individual level and would be unlikely to alter clinical decision-making for any single patient. Its public-health relevance derives chiefly from threshold effects at the population scale: given the diagnostic cut points at 5.7% and 6.5%, a shift of this size could reclassify a non-trivial fraction of individuals near these boundaries. This interpretation should be viewed as illustrative rather than prescriptive, and the findings do not support the use of smoking status as an individual-level adjustment to HbA1c-based screening.

This study has several strengths. The use of nationally representative NHANES data with complex survey weighting enhances generalizability to the U.S. adult population. Our demonstration that objective serum cotinine measurement validates self-reported smoking classification means that both can be used as exposure methods for analysis of the effects of smoking. Our sequential modeling enabled us to measure the attributes of each covariate, providing a more comprehensive analysis than previously reported. Stratification by diabetes status also revealed heterogeneity not present in the full population, and the validation of both self-reported and cotinine measures strengthens the robustness of these associations.

From a public health perspective, these findings reinforce the importance of early smoking cessation interventions. The absence of HbA1c elevation among non-diabetic former smokers, despite their higher adiposity compared to never smokers, suggests that metabolic recovery is achievable following cessation. This may provide additional motivation for smoking cessation counseling, particularly in individuals with other cardiometabolic risk factors. The use of serum cotinine to validate self-reported smoking status strengthens the evidence base and confirms that environmental tobacco smoke exposure, unlike active smoking, is not associated with measurable HbA1c changes in this population.

This study has several limitations. First, the cross-sectional design precludes causal inference. In addition, the NHANES design limited our ability to measure smoking cessation timelines or smoking intensity. Our test for interaction did not reach statistical significance; therefore, the results should be interpreted cautiously. Residual confounding from diet, physical activity, and alcohol use, which were not incorporated into our models, is also possible.

## Conclusions

In this nationally representative analysis, current smoking was statistically significantly associated with higher HbA1c among adults without diabetes, whereas former smoking was not. These associations were attenuated to the null in the full population after comprehensive adjustment for central adiposity, inflammatory markers, and lipid measures.

From a clinical perspective, the observed HbA1c elevation among current smokers without diabetes is modest in absolute terms and should not be interpreted as evidence for individual-level diagnostic adjustment. At population scale, however, even small upward shifts may reclassify some individuals near the 5.7% prediabetes threshold, particularly where smoking status is not accounted for in risk-stratification models. The absence of residual HbA1c elevation among former smokers is compatible with metabolic recovery following cessation, and together these findings support emphasizing early smoking cessation, particularly prior to the onset of established diabetes. Replication in larger, adequately powered studies with longitudinal follow-up is warranted to confirm the subgroup pattern and to characterize the time course of glycemic recovery after cessation in adults without diabetes.

## Appendices

Appendix 1

Table 5 shows the weighted percentages of missing observations from the self-reported smoking categories. 

**Table 5:** Missingness Analysis Abbreviations: HbA1c, hemoglobin A1c; HDL, high-density lipoprotein; hsCRP, high-sensitivity C-reactive protein.

Variable	Never	Former	Current
HbA1c	4.4%	3.2%	4.3%
Waist circumference	5.3%	4.5%	4.7%
Systolic blood pressure	4.0%	2.3%	3.8%
Non–HDL cholesterol	5.4%	3.9%	5.2%
log(hsCRP)	5.8%	4.1%	5.6%

Appendix 2 

A quintile analysis was reported as the primary dose-response sensitivity check (Table 6).

**Table 6:** Dose-Response Analysis - Cotinine Quintiles and HbA1c (N=9,221) Abbreviations: CI, confidence interval; HbA1c, hemoglobin A1c. *Adjusted for same variables as Model 6. †Detection limit: 0.011 ng/mL; 34% of values were at this limit, primarily in Q1.

Quintile	Cotinine Range (ng/mL)	N	β (95% CI)*	p-value
Q1 (reference)	≤0.011 (detection limit)†	1,845	0.00 (reference)	-
Q2	0.011–0.019	1,844	0.0021 (−0.0792, 0.0833)	0.957
Q3	0.019–0.066	1,844	0.0345 (−0.0337, 0.1027)	0.298
Q4	0.066–57.7	1,844	0.0012 (−0.0727, 0.0751)	0.972
Q5	57.7–1,620	1,844	0.0568 (−0.0352, 0.1488)	0.208
Trend	Per quintile increase		0.0117 (−0.0075, 0.0308)	0.217

Appendix 3

A log-transformed continuous analysis and a winsorized analysis (capped at the 99th percentile) were conducted to confirm robustness to specification choices (Table 7).

**Table 7:** Sensitivity Analysis - Winsorized Cotinine Robustness to Extreme Values (N=9,221) Abbreviations: CI, confidence interval; SE, standard error. *Adjusted for same variables as main analysis. Winsorized specification capped cotinine at 1,620 ng/mL (99th percentile)

Cotinine Specification	β (95% CI)*	SE	p-value
Standard: log(1 + cotinine)	0.0086 (−0.0053, 0.0225)	0.0066	0.210
Winsorized: log(1 + cotinine_99p)	0.0087 (−0.0053, 0.0227)	0.0067	0.207

Appendix 4

Table 8 shows the overall concordance between self-reported smoking and cotinine categories (Table 8). 

**Table 8:** Distribution of Self-Reported Smoking Status and Cotinine Categories (N=9,247) Weighted row percentages with 95% confidence intervals. Expected concordance: high cotinine in current smokers and low in never smokers. Footnote: Analysis included all participants with complete smoking and cotinine data, regardless of covariate availability (N=9,247 vs. N=9,214 in adjusted models).

Self-Reported Smoking Status	<0.05 ng/mL	0.05–<3.0 ng/mL	≥3.0 ng/mL
Never Smokers	76.0% (73.8%, 78.2%)	17.9% (15.9%, 19.8%)	6.1% (5.1%, 7.1%)
Former Smokers	60.9% (57.3%, 64.4%)	21.0% (18.4%, 23.6%)	18.1% (16.2%, 20.0%)
Current Smokers	1.5% (0.6%, 2.4%)	3.8% (2.5%, 5.1%)	94.7% (93.2%, 96.2%)
